# The ORF8 Protein of SARS-CoV-2 Modulates the Spike Protein and Its Implications in Viral Transmission

**DOI:** 10.3389/fmicb.2022.883597

**Published:** 2022-05-19

**Authors:** Jen-Mei Chou, Jo-Ling Tsai, Jo-Ning Hung, I-Hua Chen, Szu-Ting Chen, Ming-Han Tsai

**Affiliations:** ^1^Institute of Microbiology and Immunology, National Yang Ming Chiao Tung University, Hsinchu, Taiwan; ^2^Institute of Microbiology and Immunology, National Yang-Ming University, Taipei, Taiwan; ^3^College of Medicine, National Yang Ming Chiao Tung University, Hsinchu, Taiwan; ^4^Institute of Clinical Medicine, National Yang Ming Chiao Tung University, Hsinchu, Taiwan; ^5^Research Center for Epidemic Prevention, National Yang Ming Chiao Tung University, Hsinchu, Taiwan

**Keywords:** ORF8 accessory protein, SARS-CoV-2, spike protein, viral packaging, viral transmission

## Abstract

COVID-19 is currently global pandemic caused by severe acute respiratory syndrome coronavirus 2 (SARS-CoV-2). Accompanying the rapid spread of the error-prone RNA-based genome, several dominant SARS-CoV-2 variants have been genetically identified. The mutations in the spike protein, which are essential for receptor binding and fusion, have been intensively investigated for their contributions to viral transmission. Nevertheless, the importance of other viral proteins and their mutations in SARS-CoV-2 lifecycle and transmission remains fairly understood. Here, we report the strong potency of an accessory protein ORF8 in modulating the level and processing of the spike protein. The expression of ORF8 protein does not affect propagation but expression of spike protein, which may lead to pseudovirions with less spike protein on the surface, therefore less infection potential. At the protein level, ORF8 expression led to downregulation and insufficient S1/S2 cleavage of the spike protein in a dose-dependent manner. ORF8 exhibits a strong interaction with the spike protein mainly at S1 domains and mediates its degradation through multiple pathways. The dominant clinical isolated ORF8 variants with the reduced protein stability exhibited the increased capacity of viral transmission without compromising their inhibitory effects on HLA-A2. Although the increase in spike protein level and Spike pseudovirus production observed by using highly transmissible clinical spike variants, there was no significant compromise in ORF8-mediated downregulation. Because ORF8 is important for immune surveillance and might be required for viral fitness *in vivo*, the alteration of the spike protein might be an optional strategy used by SARS-CoV-2 to promote viral transmission by escaping the inhibitory effects of ORF8. Therefore, our report emphasized the importance of ORF8 in SARS-CoV-2 spike protein production, maturation, and possible evolution.

## Introduction

COVID-19, an emerging disease caused by severe acute respiratory syndrome coronavirus 2 (SARS-CoV-2), has rapidly become a worldwide pandemic that has infected more than 500 million people and is responsible for over 6 million fatalities (WHO, 2022). SARS-CoV-2 is an enveloped single-strand positive RNA virus and its genome is ranging from 26 to 32 kb in length. It encodes 14 open reading frames (ORFs), two-thirds of its genome encode 16 non-structural proteins (NSP 1–16) that comprise of the replicase complex. These NSPs play numerous roles in the replication and virus assembly processes. The remaining one-third close to 3′ end of its genome encodes nine accessory proteins, ORF3-10, and four structural proteins, including spike (S), envelope (E), membrane (M), and nucleocapsid (N). These accessory proteins and structural proteins are highly polymorphism for most SARS-related coronavirus, especially spike and ORF8 (Hu et al., 2017; Gordon et al., 2020; Harrison et al., 2020; Naqvi et al., 2020). Accompanying the fast global spread of SARS-CoV-2 and its RNA-based viral genome, many clinical variants have been reported to be highly associated with an increasing transmission ability (Korber et al., 2020; Harvey et al., 2021; Li et al., 2021; Plante et al., 2021; Washington et al., 2021; Zhou and Wang, 2021).

Mutations identified in the spike protein have been particularly highlighted because this protein can be processed into homotrimeric glycoproteins (each monomer comprising S1 and S2 subunits) on the viral envelope that are necessary for binding to receptors of host cells and cell fusion (Shang et al., 2020; Walls et al., 2020; Barrett et al., 2021). The roles of some dominant amino acid substitutions in the spike protein derived from highly transmissible SARS-CoV-2 clinical variants have implications in increasing binding affinity to the host receptor ACE2 protein, fitness in the infected hosts, immune escape, or antigen escape from neutralizing antibodies (Zhang et al., 2020; Harvey et al., 2021; Touret et al., 2021). Importantly, the spike protein is also the target recognized by most of the currently available SARS-CoV-2 vaccines; thus, alterations in the spike protein may also affect immune protection in vaccinated populations (Fenwick et al., 2021; Harvey et al., 2021; Kaku et al., 2021; Liu et al., 2021).

Moreover, other viral proteins of SARS-CoV-2 have also been found to undergo variations during the progression of COVID-19 (Toyoshima et al., 2020; Joshi et al., 2021), whereas the roles of viral transmission, pathogenicity, and viral lifecycle of each SARS-CoV-2 viral protein and variant remain not fully understood (Ceraolo and Giorgi, 2020). Among these SARS-CoV-2 viral proteins, the accessory protein ORF8, an immunoglobulin-like protein with highly immunogenic property, remains one of the most hypervariable gene evolving proteins among betacoronaviruses and exhibits the least homology with SARS-CoV viral proteins (Ceraolo and Giorgi, 2020; Lu et al., 2020; Tan et al., 2020; Zinzula, 2021). Clinically, several SARS-CoV-2 variants with ORF8 deletions show a higher transmission trend and a milder disease rate, which implies that ORF8 is not needed for viral genome replication and might play roles in pathogenesis (Gong et al., 2020; Su et al., 2020; Young et al., 2020; Zhou and Wang, 2021). Nevertheless, such ORF8-deleted SARS-CoV-2 variants have not become dominant strains worldwide in the current COVID-19 pandemic, which implies the importance of ORF8 in the human-to-human transmission of SARS-CoV-2 during its viral evolution. For example, SARS-CoV-2 might take advantage of ORF8 expression to achieve immune evasion by downregulating MHC-I and T cell cytotoxicity or immune modulation (Park, 2020; Fahmi et al., 2021; Lin et al., 2021; Rashid et al., 2021; Zhang et al., 2021), which might facilitate the incubation period of viral replication in infected individuals and thus contribute to transmission. However, whether ORF8 directly participates in viral transmission should be carefully investigated.

In this study, we found that the expression of the accessory protein ORF8 strongly inhibited the production of SARS-CoV-2 Spike pseudoviral particles in HEK293T cells. The expression of ORF8 strongly inhibited the protein level and S1/S2 cleavage of spike proteins derived from different spike variants in a dose-dependent manner. Mechanistically, we observed that ORF8 interacted with the spike protein strongly at S1 domain and might contribute to modulation of the spike protein through multiple mechanisms. ORF8 variants of dominant clinical samples showed less potency in modulating the spike protein by reducing their stability without compromising the inhibition of MHC-I. Ultimately, we observed that the inhibitory effects of the ORF8 protein affected the spike protein with mutations observed in highly transmissible SARS-CoV-2 variants. Taken together, these results show the strong potency of the ORF8 protein in modulating the spike protein and may consequently influence the efficiency of the packaging of spike-bearing viral particles.

## Materials and Methods

### Cell Lines

HEK293 cells are derived from human embryonic kidney cells (ATCC: CRL-1573), and the HEK293T cell line is a derivative of HEK293 cells that contains the SV40 T-antigen (ATCC: CRL-3216). HEK293T cells stably expressing human ACE2 (HEK293T/hACE2) were obtained after transduction with lentiviruses carrying hACE2 and blasticidin resistance genes and selected with 10 μg/ml blasticidin (Invitrogen). HEK293 cells stably expressing the spike protein (HEK293/Spike) were transduced with lentiviruses carrying spike and neomycin resistance genes and selected with 400 μg/ml G418 (Invitrogen). All cells used in this study were maintained in RPMI medium (Invitrogen) supplemented with 10% fetal bovine serum (FBS, HyClone) in a humidified cell culture incubator at 37°C.

### Plasmids and Oligonucleotides

pGBW-m4133655 and pGBW-m4134293 are mammalian expression plasmids for SARS-CoV-2 ORF8, and ORF3a protein with a 3XFLAG tag at their C-terminus was a gift from Ginkgo Bioworks and Benjie Chen (Addgene plasmids #151978 and #152658). pcDNA3.1-SARS2-Spike is an expression plasmid carrying the wild-type SARS-CoV-2 spike protein with a C9 tag at its terminus and was a gift from Fang Li (Addgene plasmid #145032). pLAS2 is a lentiviral vector with the CMV promoter and was obtained from the RNAi core of Academia Sinica, Taiwan. We therefore inserted an IRES-EGFP cassette after the CMV promoter of the pLAS2 lentiviral plasmid (pLAS2-IRES-EGFP). Therefore, the ORF8 and ORF3a genes were subcloned into pLAS2-IRES-EGFP by PCR cloning, and the pLAS2-ORF3a-IRES-EGFP and pLAS2-ORF8-IRES-EGFP plasmids were then constructed. All ORF8 mutations were created through overlapping PCR using the ORF8 plasmid obtained from Addgene #151978 as a template and subcloned into pLAS2-IRES-EGFP, which included S24L, V62L, L84S, C20A, and del73-76. Expression plasmids carrying important spike protein clinical variants, namely, pcDNA3.1-SARS2-Spike with D614G or D614G + N501Y mutations, were generated using pcDNA3.1-SARS2-Spike (Addgene plasmid #145032) as the backbone and modified by PCR cloning. Expression plasmid carrying the spike protein D614G + N501Y was applied for most of the transfection and pseudovirus production experiments and few exceptions were carefully annotated. A series of spike mutations or deletions were constructed using pcDNA3.1-SARS2-Spike D614G/N501Y and restriction enzyme digestion or overlapping PCR, which included GSAS mutations, L18F/T20N/P26S, and T19R, and the deletion mutations with the indicated amino acids were 586–978, 820–1,273, 35–102, 295–639, 306–527 (RBD domain), 528–685 (SD1/2 domain), 14–685 (N-terminal deletion, Ndel, S1 deletion), and HV 69–70. We further constructed a plasmid using pcDNA3.1-SARS2-Spike D614G/N501Y as the backbone, but its C9 tag at the C-terminus was removed by PCR. The sequences of the plasmids constructed by PCR cloning were further validated through Sanger sequencing. For the constructs performed by restriction enzyme digestion: pcDNA3.1-SARS2-Spike with deletion at 586–978 amino acids was constructed by the digestion of pcDNA3.1-SARS2-Spike D614G + N501Y with EcoRV restriction enzyme and performed self-ligation; pcDNA3.1-SARS2-Spike with deletion at 35–102 amino acids was constructed by the digestion of pcDNA3.1-SARS2-Spike D614G + N501Y with SacII restriction enzyme and performed self-ligation; pcDNA3.1-SARS2-Spike with deletion at 295–639 amino acids was constructed by the digestion of pcDNA3.1-SARS2-Spike D614G + N501Y with BamHI restriction enzyme, filled in the perturbing ends with Klenow enzyme, and perform self-ligation. The lenti-ACE2 plasmid and a synthesized codon-optimized spike gene constructed in pCDNA3.1 were gifts from Academia Sinica, Taiwan. All synthesized oligonucleotides are listed in Supplementary Table 1.

### Transfections

All transfection experiments were performed with PEI MAX (MW 40,000, Polysciences 24765-1) following the manufacturer’s instructions.

### Production of Pseudovirions and Cell Infection

Pseudovirions were produced by the cotransfection of HEK293T cells with pCMVR8.91, pLAS2-based lentiviral plasmids, and plasmids encoding either SARS-CoV-2 S or VSV-G at the ratio 6.25: 6.5: 1.1 using PEI transfection. The supernatants were collected at 60 h post-transfection and passed through a 0.45-μm filter. In some experiments, the SARS-CoV-2 Spike pseudovirions were further purified from the supernatant by sucrose centrifugation described by Jiang et al. (2015) and resuspended in culture medium. To evaluate the transduction efficiency of each pseudoviral sample, 5,000 HEK293T/hACE2 cells were seeded in 96-well plates per well and inoculated with different volumes of pseudovirions. The transduction efficiency of each pseudoviral sample was evaluated 96 h post-transduction by analyzing the percentage of GFP-positive cells and documented using a fluorescence microscope (Zeiss) or quantified by flow cytometry (FACSCalibur).

### Antibodies

A rabbit polyclonal antibody against the SARS-CoV-2 spike protein (obtained from Academia Sinica, Taiwan) was used for all immunofluorescence staining, flow cytometry, and immunoblotting assays of the spike protein. Primary mouse monoclonal antibodies against FLAG tag (Sigma-Aldrich F1804), HLA-A2 (BioLegend 343302), HLA-A,B,C (BioLegend 311402), B2-microglobulin (BioLegend 316302) were used. Beta actin (Sigma-Aldrich A5441) and HSP90 (OriGene TA500494) were used in all the experiments. The secondary antibodies used for immunofluorescence staining or flow cytometry were goat anti-mouse coupled to Cy3 (Invitrogen A10521) or Alexa Fluor 647 (Invitrogen A21244), goat anti-rabbit coupled to Cy3 (Invitrogen A10520) or Alexa Fluor 647 (Invitrogen A32733), and Donkey anti-rabbit coupled to PE (BioLegend 406,421). Horseradish peroxidase-coupled goat anti-mouse or rabbit antibodies (Jackson Laboratory) were applied as secondary antibodies in the Western blot analyses.

### Binding Assay

The pseudovirus samples were incubated with 20,000 HEK293T/hACE2 cells under constant rolling at 4°C. After 1 h of incubation, the cells were collected by centrifugation, washed three times with PBS, and dropped on glass slides. The bound SARS-CoV-2 Spike pseudovirions or free-form spike proteins in the culture supernatant were thus visualized after immunostaining.

### Immunostaining

Cells dried on glass slides or cultured in a glass culturing chamber were fixed with 4% paraformaldehyde in PBS for 20 min at room temperature and then permeabilized in PBS with 0.5% Triton X-100 for 2 min except for the samples used in the binding assay. The cells were thus incubated with primary antibody for 30 min at 37°C, washed three times in PBS, and incubated with secondary antibody conjugated to Cy3 or Alexa Fluor 647 for 30 min at 37°C. After three washes with PBS, the slides were embedded in 90% glycerol in PBS.

### Western Blotting

Proteins were extracted using standard lysis buffer [150 mM NaCl, 0.5% NP-40, 1% sodium deoxycholate, 0.1% SDS, 5 mM EDTA, 20 mM Tris–HCl pH 7.5, and proteinase inhibitor cocktail (Roche)] for 15 min on ice and then sonicated to shear the genomic DNA. The lysates were clarified by centrifugation and denatured in Laemmli buffer for 10 min at 95°C. For some experiments, if non-denaturing conditions were needed for the samples, the lysates were mixed with Laemmli buffer without 2-mercaptoethanol. The proteins were then separated on SDS–polyacrylamide gels and blotted onto a PVDF membrane (Bio-Rad). The blots were first blocked with 3% milk in PBST (PBS with 0.05% Tween 20) and then incubated with primary antibody against the candidate protein at room temperature for 1 h. After extensive washes with PBST, the blot was incubated for 1 h with secondary antibodies. The bound antibodies were visualized with a chemiluminescent reagent (Pierce). The relative intensity of the signals obtained after Western blotting were quantified by the ImageJ software. For evaluating the expression level of spike protein, all fragments of spike protein visualized above 100 kDa (containing the cleaved S1/S2 fragments and the full-length spike protein) were selected for quantification.

### Co-immunoprecipitation

The cells were washed three times with PBS and lysed in lysis buffer (20 mM Tris–HCl pH 8.0, 137 mM NaCl, 1% Nonidet P-40, and 2 mM EDTA) at 4°C under rotation at 150 rpm for 1 h. The lysate was centrifuged at 21,000 ×*g* for 10 min, and the clarified lysates were collected and subjected to immunoprecipitation using a Dynabeads Protein G Immunoprecipitation kit (Invitrogen 10007D) following the manufacturer’s instructions. We applied a rabbit polyclonal antibody against the SARS-CoV-2 spike protein and a mouse monoclonal antibody against the FLAG tag for immunoprecipitation. After the incubation of lysates with antibody-loaded Protein G Dynabeads at room temperature for 1 h, the beads together with the pulled down proteins were collected using a magnet and washed four times with washing buffer. The immunoprecipitates together with Dynabeads Protein G were then eluted by boiling in Laemmli buffer for 10 min at 95°C and separated on SDS–polyacrylamide gels for Western blotting. All HEK293T cells in the samples used for immunoprecipitation experiments were cotransfected with the spike-encoding plasmid and the ORF8-, or ORF3a-encoding plasmid, or control plasmid (EV) at a ratio of 1–9.

### Inhibitors of Protein Degradation

Cells were treated with the following inhibitors for 24 h and subjected for flow cytometry or immunoblotting. DMSO (Sigma D8418), MG132 (Cayman 13,697), DBeQ (Cayman 15,318), bafilomycin A1 (Baf A1; Cayman 11,038), chloroquine (Cayman 14,194), and NH_4_Cl (Sigma A9434).

### Flow Cytometry

The cells were washed three times with PBS and then incubated in ice-cold staining buffer (PBS with 10% heat-inactivated goat serum) for 20 min. The cells were stained with primary and secondary antibodies diluted in staining buffer accordingly and analyzed with a FACSCalibur instrument. The data were further analyzed using FlowJo software.

### Inhibition of Protein Translation by Cycloheximide Treatment

Cells after 24 h post-transfection were treated with cycloheximide (Syrusbioscience 101-66-81-9) at 100 μg/ml at the indicated time points and subjected for Western blotting.

### Statistical Analysis

The differences between two groups were compared by Student’s *t*-tests using GraphPad Prism 5. *p* < 0.05 indicates a significant difference. The relative expression levels of the spike protein and MHC-I obtained by flow cytometry were quantified using FlowJo software, and the mean fluorescence intensity (MFI) values were used for statistical analysis if needed.

## Results

### The Expression of ORF8 Abrogates the Production of SARS-CoV-2 Spike Pseudovirions

**Figure 1:**
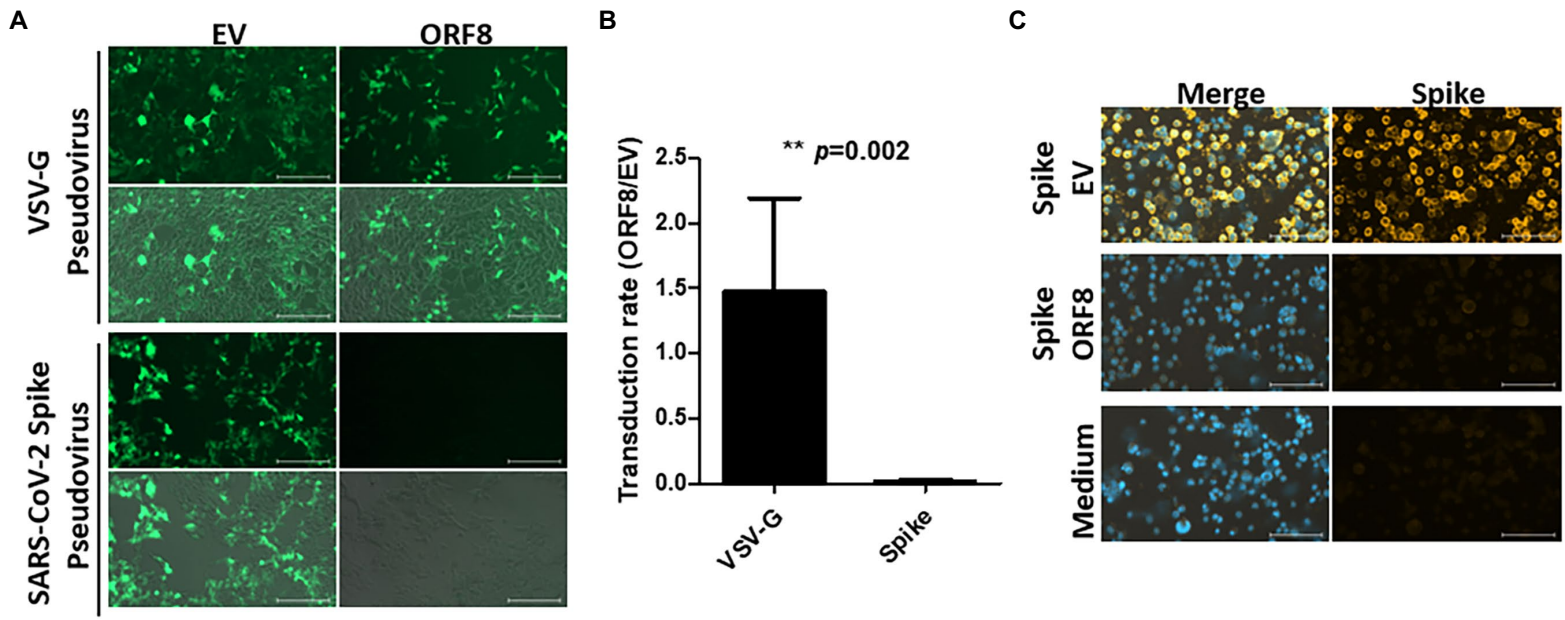
ORF8 production abrogates the production of SARS-CoV-2 Spike pseudovirus. VSV-G and SARS-CoV-2 Spike pseudoviruses were produced in HEK293T cells by transfecting plasmids encoding Gag-pol or envelope protein (VSV-G or spike) and package plasmids encoding IRES-GFP (EV) or ORF8-IRES-GFP (ORF8). The same amount of viral supernatant from each sample were assayed to determine its transduction efficiency using HEK293T/hACE2 cells. The transduction efficiency were measured by fluorescence microscopy and flow cytometry 4 days after transduction. **(A)** Representative fluorescence microscopy data. For each result, the upper figure shows the GFP signal only and the lower figure shows the merged figure combined with the photo obtained by phase contrast. **(B)** The transduction rate of pseudoviruses prepared with ORF8 vs. control plasmids were quantified by flow cytometry from 5 independent experiments. **(C)** The purified Spike pseudoviruses were exposed to HEK293T/hACE2 cells on ice with rolling for 1 h. After extensive washes with PBS to remove the unbound viral particles, the bound viral particles were visualized using an antibody against the spike protein with a Cy3-labeled secondary antibody; DAPI were applied for nuclear staining. The data are shown as the means ± SDs (error bars). Student’s *t*-test was used, and *p* < 0.05 indicates a statistically significant difference; ** *p* < 0.01. The scale bar in **(A)** represents 200 μm and in **(C)** represents 100 μm.

To address whether ORF8 influences functional SARS-CoV-2 virion production, we first used a pseudovirus system that has been intensively applied to study the functionality of the spike protein in SARS-CoV-2 studies (Ou et al., 2020; Shang et al., 2020). To establish this system, a lentiviral plasmid with cistronic expression of ORF8 and GFP protein was constructed, and the same lentiviral plasmid with only the GFP protein served as a control in the later experiments. This design allowed the evaluation of pseudoviral transduction efficiency through GFP-expressing cells. These two lentiviral plasmids were applied for the production of VSV-G or SARS-CoV-2 Spike lentiviral pseudovirions in HEK293T cells, and the infectivity of these pseudovirions was evaluated by calculating the percentage of GFP-positive cells after the transduction of HEK293T cells stably expressing human ACE2 (HEK293/hACE2). At day 4 post-transduction, we observed that VSV-G pseudovirions produced by both lentiviral plasmids exhibited a similar transduction efficiency (Figures 1A,B). Moreover, HEK293/hACE2 cells could only be transduced by SARS-CoV-2 Spike pseudovirions produced by lentiviral plasmids without the ORF8 gene (Figures 1A,B). Since VSV-G lenti-based pseudovirus exhibits the pantropic infectivity toward different mammalian cells, our results suggested that ORF8 did not impede the propagation of lentiviral pseudoviruses but had significant impact on the transduction efficiency of the Spike pseudovirions in HEK293/hACE2 cells. To elucidate whether ORF8 participates in the packaging of SARS-CoV-2 Spike pseudovirions, the viral particles were first enriched through gradient centrifugation to remove the free-form spike protein complexes, and concentrated SARS-CoV-2 Spike pseudovirions were used in the viral binding assay. By exposing HEK293/hACE2 cells to SARS-CoV-2 Spike pseudovirions and visualizing the bound viral particles by immunofluorescence staining with a spike-specific antibody, we confirmed that a substantial amount of SARS-CoV-2 Spike pseudovirions could be produced in our system through a control lentiviral plasmid, whereas SARS-CoV-2 Spike pseudovirions could barely be observed if the ORF8 protein was expressed in the viral packaging cells (Figure 1C). Interestingly, we observed that ORF8 expression still allowed the production of viral particle-free spike protein in the culture supernatant through a binding assay (Supplementary Figure 1). Taken together, these data showed that the ORF8 protein has a strong impact on the packaging of SARS-CoV-2 Spike pseudovirions.

### ORF8 Expression Leads to Downregulation of the Spike Protein and Impedes Its S1/S2 Processing

**Figure 2:**
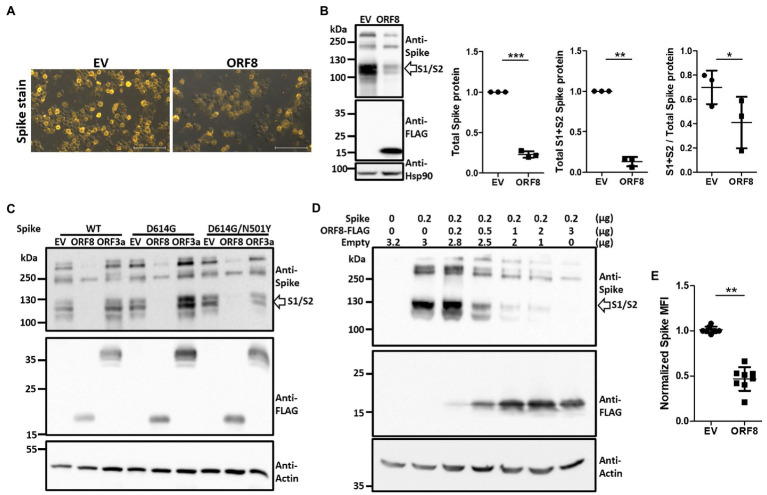
Expression of ORF8 shows the downregulation and insufficient S1/S2 processing of the spike protein in a dose-dependent manner. **(A)** Spike protein immunostaining of Spike pseudovirus-packaging cells was performed. One representative result is shown, and the yellow color indicates the spike signal. EV: empty vector. **(B)** Immunoblots of Spike pseudovirus-packaging cells were obtained with antibodies specific to the spike protein and FLAG tag to visualize the protein level of the spike protein and ORF8. An antibody specific to Hsp90 served as a loading control. The scatter plot shows the relative intensity of the signals quantified by the ImageJ software from three independent experiments. The cleaved S1 and S2 fragments (between 100 to 130 kDa) were selected and determined as “S1 + S2 spike protein” and all fragments visualized above 100 kDa (containing the cleaved S1/S2 fragments and the full-length spike protein) were all selected and evaluated as “total spike protein” for quantification in this figure. In order to visualize the spike signal in ORF8-transfected samples together with EV-transfected samples in the same blot, the intensity of S1/S2 fragment of EV-transfected might reach plateau level then the real intensity might be underestimated. So the true efficiency of S1/S2 cleavage in EV-transfected cells might be much higher than the data shown here. **(C)** Immunoblots of cells transfected with ORF8 or ORF3a in combination with different spike variants. Here, an antibody specific to the FLAG tag indicates the protein levels of ORF8 and ORF3a. WT: Spike obtained from the original Wuhan strain; D614G: WT spike protein with the D614G mutation; D614G/N501Y: WT spike protein with the D614G and N501Y mutations. **(D)** Immunoblots of HEK293T cells transfected with different amounts of ORF8-expressing plasmids were obtained 3 days post-transfection. Here, an antibody against actin served as the loading control. Empty: empty vector does not encode ORF8. **(E)** FACs analyses of surface spike protein were performed using HEK293T cells cotransfected with spike-encoding plasmid together with pLAS2-based plasmid carrying ORF8 or empty vector at 1–9 ratio at 3 days post-transfection. The data are shown as the means ± SDs (error bars). Here, the paired Student’s *t*-test was used, and *p* < 0.05 indicates a statistically significant difference; *p < 0.05, **p < 0.01, ***p < 0.001. The scale bar in each figure represents 100 μm.

To elucidate the mechanism through which ORF8 impedes the packaging of SARS-CoV-2 spike-bearing pseudovirions, we analyzed the expression level of the spike protein in these pseudoviral packaging cells through immunofluorescence staining. The overexpression of ORF8 in these cells significantly downregulated spike protein expression (Figure 2A). The expression level and protein processing of the spike protein in these cells were further investigated through immunoblotting, and the results showed that ORF8 expression not only influenced the expression level of the spike protein but also affected the efficiency of the S1/S2 cleavage of this protein (Figure 2B). To further exclude the possibility that a reduction in spike protein expression was due to competition with transcription factors during the cotransfection of two plasmids into the cells, we further constructed another plasmid encoding ORF3a, which is an accessory protein of SARS-CoV-2, into the same lentiviral plasmid as an additional control for comparison. Our results confirmed that ORF8- but not ORF3a-overexpressing cells strongly reduced the expression and cleavage of the S1/S2 fragment of the spike protein (Figure 2C). We also confirmed that the same phenomenon could be observed using different spike protein variants (Figure 2C) and not due to the unwanted artifact causing by C9-tag fused with spike protein (Supplementary Figure 2A). By using a non-reducing gel, we further confirmed the previously reported dimerization of the ORF8 protein that might be potentially mediating immune suppression (Flower et al., 2021; Supplementary Figure 2B).

To further determine whether the impact of ORF8 on the spike protein can be observed in non-lentiviral-producing cells, we transiently transfected HEK293T cells with a plasmid encoding the ORF8 gene together with a plasmid encoding the spike gene at different ratios. Our results confirmed that ORF8 protein can inhibit the expression level and S1/S2 processing of the spike protein in a dose-dependent manner (Figure 2D). Finally, we also observed the reduced expression of the surface spike protein in ORF8-transfected cells through flow cytometry (Figure 2E).

### ORF8 Protein Interacts With S1 Domain of the Spike Protein

**Figure 3:**
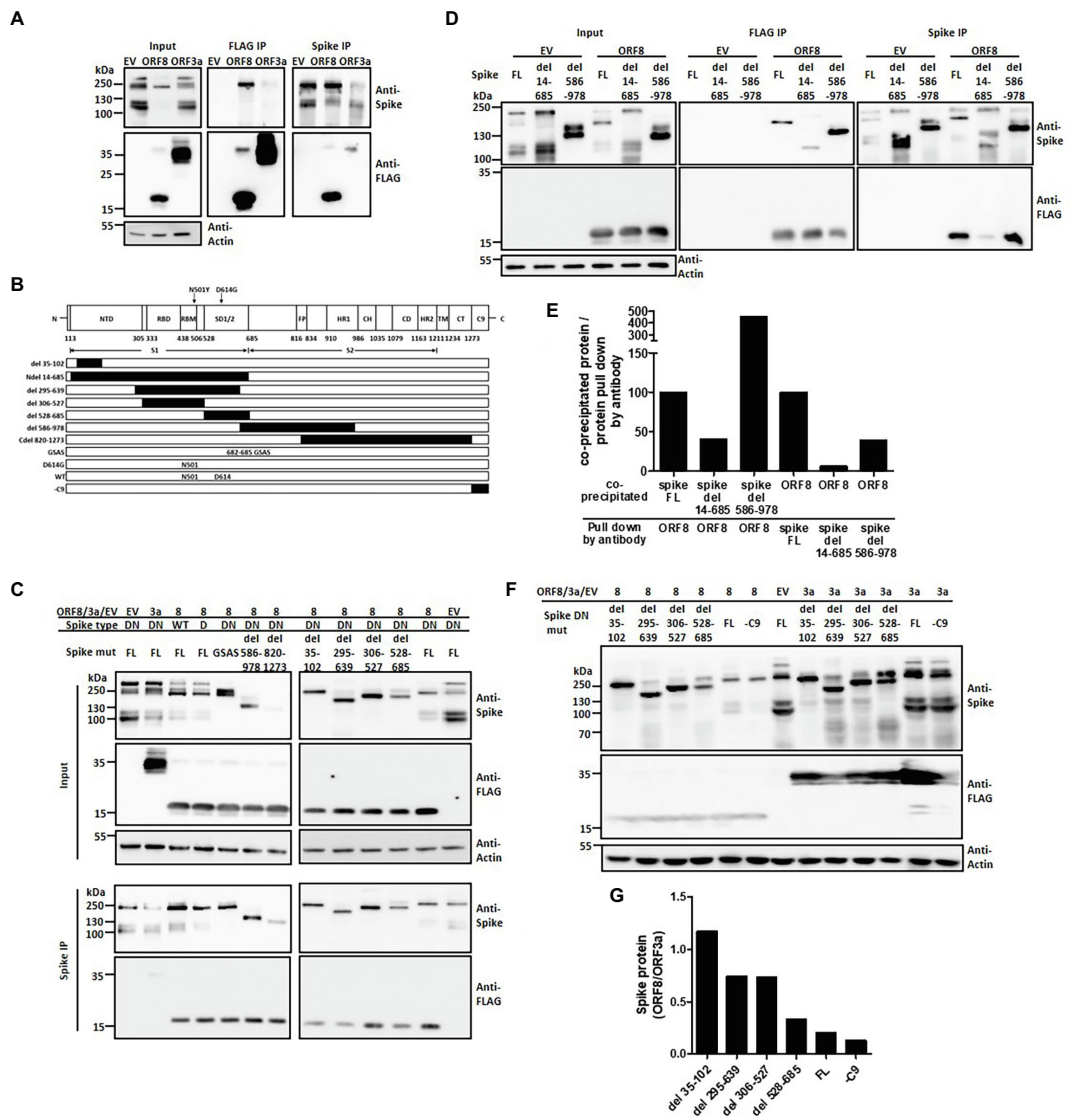
ORF8 interacts with the spike protein at different domains. HEK293T cells were cotransfected with the plasmids described in each figure, and the cell lysates were prepared and subjected to immunoprecipitation using an antibody against the FLAG tag or spike protein. In all following immunoprecipitation results, immunoblots were obtained using total cell lysates (input), and the proteins were pulled down by Protein G Dynabeads conjugated with the indicated antibody (IP). **(A)** Representative immunoprecipitation data obtained using either anti-FLAG or anti-spike antibodies are shown. EV: empty vector. **(B)** A series of spike proteins with different mutations or deletions were constructed for the experiments described in the cartoon figure. Schematic figure presenting the domain arrangement of the spike protein was adapted from Duan et al. with few modifications. NTD: N-terminal domain; RBD: receptor-binding domain; RBM: receptor-binding motif; SD1/2: subdomain 1 and 2; FP: fusion peptide; HR1 and HR2: heptad repeat 1 and 2; CH: central helix; CD: connector domain; TM: transmembrane domain; CT: cytoplasmic tail; C9: C9 tag. The black boxes shown in this figure represent the deleted regions of the spike protein. **(C)** A series of immunoprecipitation experiments focusing on different spike variants or deletions were performed using a polyclonal anti-spike antibody. **(D)** An immunoprecipitation experiment was performed using the full-length or the mutated spike protein with deletion of the S1 (del 14-685) or S2 region (del 586-978) was shown. **(E)** Meanwhile, the intensity of interaction between ORF8 with different mutations of the spike proteins shown in **(D)** was evaluated by the evaluation of the ratio between the protein level of the co-precipitated protein vs. the protein directly pull down by the indicated antibody. **(F)** The spike protein expression levels were compared between ORF8- and ORF3-transfected cells by using immunoblotting and **(G)** the spike protein expression level in between both groups was evaluated by using the ImageJ software. Abbreviations of the spike mutants shown in **(C–F)** are as following: DN: D614G/N501Y; D: D614G; GSAS: point mutations created at 682–685 amino acids; FL: full length; -C9: spike protein without C9 tag at its C-terminal. And del 14-685, del 586-978, del 35-102, del 295-639; del 306-527; del 528-685 represent the deletion regions of the spike protein.

We therefore wish to investigate the possible mechanism through which ORF8 regulates the expression and processing of the spike protein. Through immunofluorescence staining of these two proteins in transfected HEK293T cells, we observed that the expression level of ORF8 protein seemed negatively correlated with that of the spike protein (Supplementary Figure 3). We further wished to investigate the interaction between ORF8 and spike proteins. Immunoprecipitation data showed that the ORF8 protein bound to the spike protein directly (Figure 3A). Using ORF3a as an additional experimental sample, we also observed a weak interaction between ORF3a and the spike protein (Figure 3A), as previously reported (Tan, 2005; Rihn et al., 2021). Furthermore, we would like to investigate the possible ORF8-binding site in the spike protein. To achieve this aim, we constructed a series of plasmids encoding spike mutations or deletions (Duan et al., 2020; Figure 3B). S1/S2 cleavage site is defined at 685 amino acid where the polybasic cleavage motif (RRAR) is recognized by furin protease. We examined the binding ability of ORF8 to different spike proteins with deletions in the S1 or S2 region (Figure 3C). We observed that certain spike mutants with deletion at S1 fragment 35–102, 295–639, or 528–685 amino acids showed weaker interaction with ORF8 protein by using immunoprecipitation assay. In parallel, we did not observe the reduced interaction of the spike mutations with deletions mainly at S2 fragments (586–978 and 820–1,273; Figure 3C). Indeed, the spike protein with completed S1 deletion dramatically lost the binding ability with ORF8 had also be observed (Figures 3D,E). Meanwhile, we observed that the spike proteins with deletion at N-terminal regions were less sensitive to ORF8-induced downregulation by comparing to the expression level of the spike proteins in the cells overexpressing ORF3a (Figures 3F,G).

### ORF8-Mediated Spike Protein Downregulation Is the Result of Multiple Protein Degradation Pathways

**Figure 4:**
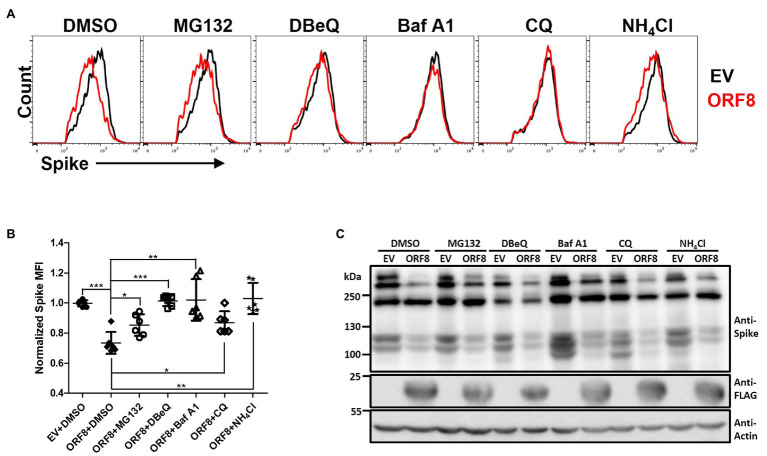
ORF8 mediates the downregulation of the spike protein through multiple pathways. **(A,B)** HEK293T cells were cotransfected with the spike-encoding plasmid together with the pLAS2-ORF8-IRES-GFP (ORF8) or pLAS2-IRES-GFP (EV) plasmid at a ratio of 1–3. Forty-eight hours after transfection, the cells were treated with DMSO, MG132 (20 μM), DBeQ (15 μM), bafilomycin A1 (Baf A1; 200 nM), chloroquine (20 μM), and NH_4_Cl (20 mM) for 24 h, and the cells were collected for flow cytometry using antibodies against the spike protein and HLA-A2. By using this method, we were allowed to analyze the cells with successful transfection by gating the GFP-positive cell population. Here, the results of the spike protein are shown as **(A)** histograms comparing EV- or ORF8-transfected cells of one experiment and **(B)** the scatter plot figure of the results from six-independent transfection. **(C)** Immunoblots of cell lysates prepared from one experiment using antibodies against the spike protein, FLAG tag, and actin. The bar figure in **(B)** shows the means ± SDs (error bars) and unpaired Student’s *t*-test was used, and *p* < 0.05 indicates a statistically significant difference; *p < 0.05, **p < 0.01, ***p < 0.001.

The physical interaction between ORF8 and spike proteins implies that ORF8 may regulate the level or processing of the spike protein through post-translational regulation. The cells were treated with various inhibitors that block the degradation of proteins through different pathways, including MG-132, which blocks the ubiquitin-proteasome system, N2,N4-dibenzylquinazoline-2,4-diamine (DBeQ), which blocks ER-associated protein degradation (ERAD), bafilomycin A1 (Baf A1) and chloroquine (CQ), which block autophagosome–lysosomal degradation, and ammonium chloride (NH_4_Cl), which blocks phagosome–lysosome fusion. After treatment of the cells with these inhibitors for 24 h, we evaluated the expression of the spike protein by flow cytometry (Figures 4A,B; Supplementary Figures 4A,C). Our data showed that the expression level of the spike protein on the cell surface could be partially rescued in ORF8-expressing cells treated with all these inhibitors at certain degree. In parallel, we also evaluated the expression level of HLA-A2 in these transfected cells since HLA-A2 had been validated can be downregulated by ORF8 (Zhang et al., 2021). We confirmed that ORF8 overexpression reduced the expression level of HLA-A2 expression in the transfected cells. Meanwhile, we also observed the similar HLA-A2 expression in between EV- and ORF8-transfected cells at this time after the treatment of bafilomycin A1 (Supplementary Figures 4B,D), which were in line with the paper published previously (Zhang et al., 2021). However, the overall spike protein recovery determined by immunoblotting was hardly observed in this experiment (Figure 4C). It is important to note that the data shown in Figure 4 and Supplementary Figure 4 were collected using cells transfected with ORF8- and spike-encoding plasmid at a lower ratio of 3:1. We could not observe the recovery of the spike protein after the treatment of inhibitors in the cells transfected with ORF8- and spike-encoding plasmid at a high ratio (9:1), possibly due to the strong downregulating effects of ORF8 on the spike protein under this condition (Figure 2D). We had tried to test the combination of different inhibitors but unfortunately high cell toxicity was observed. Overall, these data suggest that ORF8 may modulate spike protein degradation *via* multiple protein degradation pathways.

### Impact of ORF8 Mutations on the Expression Level and S1/S2 Processing of Spike Protein

**Figure 5:**
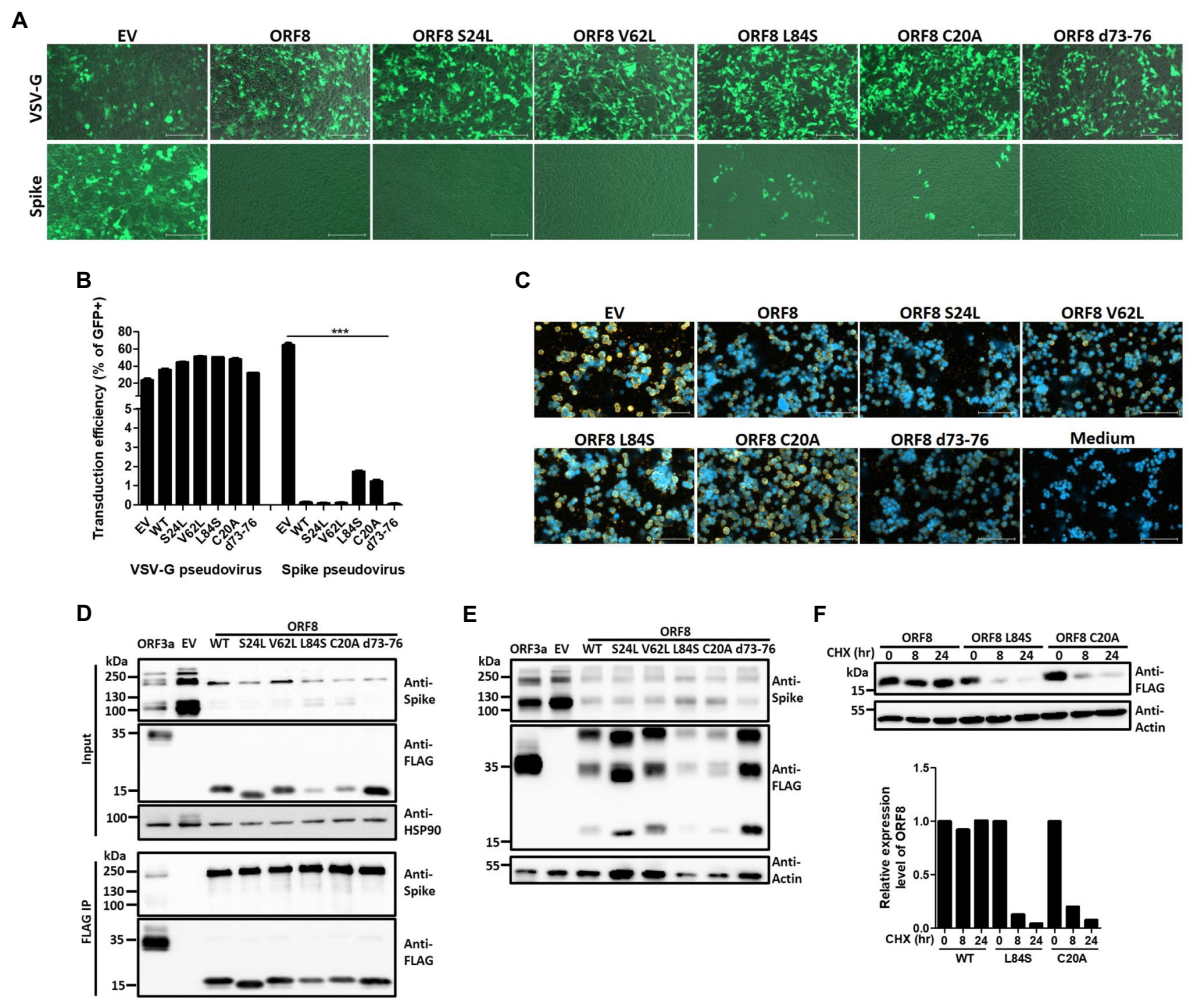
Impact of ORF8 mutations on regulating the spike protein and their own stability. Spike or VSV-G pseudoviruses were produced using the pLAS2-IRES-GFP (EV) lentiviral vector or the same vector carrying different ORF8 mutants shown as mutation sites. After the purification of these pseudoviruses, an equal amount of viral supernatant was used to test their transduction efficiencies in HEK293T/hACE2 cells, which were observed as GFP-positive cells 4 days post-transduction and were **(A)** visualized by fluorescence microscopy and **(B)** quantified by flow cytometry. EV: empty vector; WT: wild type. **(C)** The relative quantity of Spike pseudoviruses in these samples was evaluated by a binding assay of HEK293T/hACE2 cells exposed to the same volume of different viral samples, and samples were then visualized with a polyclonal antibody recognizing the spike protein and a Cy3-conjugated secondary antibody. DAPI was used for staining nuclei. **(D)** Co-immunoprecipitation assays were performed using HEK293T cells cotransfected with spike-encoding plasmid together with pLAS2-based plasmid carrying different ORF8 mutants 3 days post-transfection. Here, a polyclonal antibody against the spike protein was used. **(E)** A set of Samples similar as **(D)** was prepared in non-denature condition and subjected to immunoblotting experiment. **(F)** A cycloheximide-tracing experiment was performed with cells transfected with a plasmid encoding wild-type ORF8, L84S, or C20A mutation. The transfected cells were treated with 100 μg/ml cycloheximide at 24 h post-transfection. Immunoblots of the samples collected from the indicated time points after cycloheximide treatment were performed using an anti-FLAG antibody. Antibody against actin served as a control. The protein stability of each ORF8 mutant shown in was evaluated by comparing the protein amounts of ORF8 and actin quantified using the ImageJ software. The bar figure in **(B)** shows the means ± SDs (error bars). The unpaired Student’s *t*-test was used, and *p* < 0.05 indicates a statistically significant difference; ***p < 0.001. The figure shows the statistical analyses of the comparisons of EV vs. all ORF8 variants, WT vs. L84S, and WT vs. C20A. The scale bar in **(A)** represents 200 μm and **(C)** represents 100 μm.

The ORF8 gene encodes a rapidly evolving protein of SARS-CoV-2, and many variants of this protein have been reported (Alkhansa et al., 2021; Fahmi et al., 2021; Hussain et al., 2021; Pereira, 2021; Zinzula, 2021). A recent X-ray crystallography study of ORF8 reported found that SARS-COV-2 ORF8 can form dimers or unique large-scale assemblies through Cys20 and 73YIDI76 motifs (Flower et al., 2021). To investigate the impact of ORF8 mutants on modulating spike protein expression and cleavage, we constructed several ORF8 mutants previously found in clinical samples, including proteins with S24L, V62L, and L84S point mutations, as well as ORF8 mutants that may be important for protein structure or dimerization, including proteins with C20A and 73YIDI76 deletion, into the same lentiviral vector as that used for pseudovirion packaging. By evaluating the transducing ability of HEK293T/hACE2 cells by the SARS-CoV2 Spike lentiviral pseudovirions showing the expression of different ORF8 mutant proteins, we found that the cells expressing the ORF8 proteins with L84S or C20A mutations had significantly higher transduction rates than the other ORF8 mutants (Figures 5A,B) and the former cells also showed increased production of Spike pseudoviral particles (Figure 5C). To study the impact of these ORF8 mutants on the expression level and S1/S2 cleavage of the spike protein, HEK293T cells were cotransfected with a plasmid encoding the spike protein together with different ORF8-encoding plasmids. Immunoblot analyses of these cell lysates showed that all tested ORF8 variants and mutants strongly inhibited the expression level of the spike protein as well as the processing of the spike protein into the S1/S2 fragment (Figure 5D). However, the ORF8 L84S and C20A mutations were associated with weaker inhibition of S1/S2 cleavage, and the protein expression levels of these two mutants were also lower than those of the other ORF8 proteins (Figure 5D). We further performed immunoprecipitation of ORF8 proteins using an anti-FLAG antibody and confirmed that all these ORF8 variants and mutants exhibited strong physical interactions with the spike protein (Figure 5D). To further elucidate the influence on the spike protein by L84S, C20A, and wild-type ORF8, HEK293 cells were transfected with spike- and different ORF8-encoding plasmids at different ratios. Our data showed that the modulation of the spike protein by ORF8 L84S and C20A mutants in cells expressing these proteins at similar levels was similar to that obtained with wild-type ORF8 (Supplementary Figure 5A). Meanwhile, we did not observe any difference in the dimerization and accumulation of ORF8 protein between different variants and mutations (Figure 5E). To elucidate whether ORF8 L84S and C20A mutations acquired lower protein expression levels in the transfected cells was due to the higher protein turnover rate, we applied cycloheximide, an inhibitor of protein synthesis, to the transfected cells. The cycloheximide-tracing experiment showed that the ORF8 L84S and C20A mutants exhibited higher turnover rates than wild-type ORF8, which explained the lower protein levels of these ORF8 mutants (Figure 5F). We also examined the impact of ORF8 mutations on the downregulation of surface spike protein and MHC-I and did not observe defects with any of the tested mutants (Supplementary Figures 5B,C).

### Impact of ORF8 on Spike Protein With Mutations Identified From Highly Transmissible Clinical Variants

**Figure 6:**
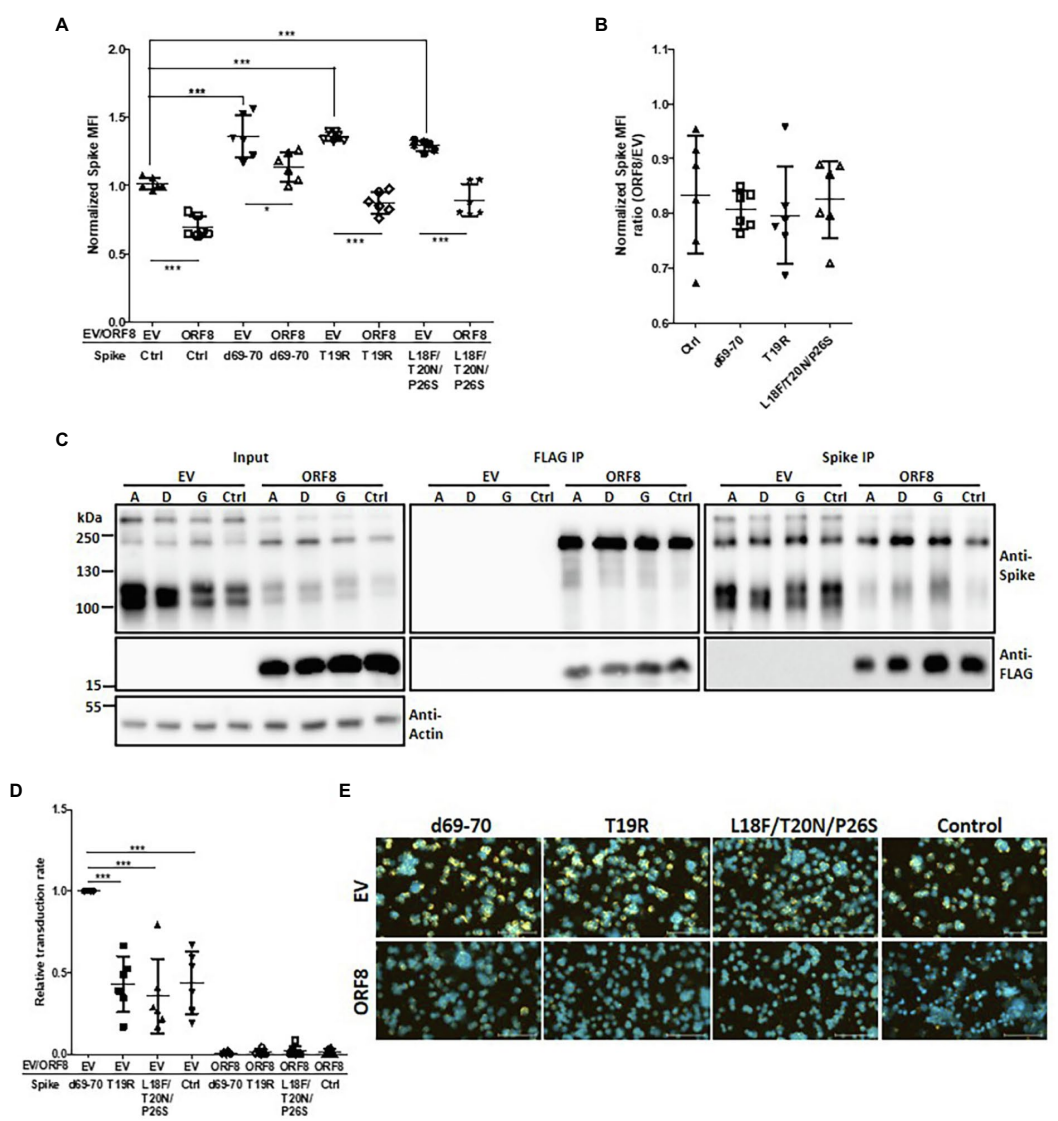
Influence of spike mutations on ORF8-mediated downregulation of the spike protein. **(A)** HEK293T cells were cotransfected with the different spike-encoding plasmid together with the pLAS2-ORF8-IRES-GFP (ORF8) or pLAS2-IRES-GFP (EV) plasmid. Three days after transfection, the cells were collected for flow cytometry using antibodies against the spike protein. Here the results were collected from six-independent experiments after normalization. Ctrl: control spike plasmid without the indicated modifications. **(B)** The same results of **(A)** but shown as the ratio between ORF8 and EV groups. No significant difference was observed. **(C)** HEK293T cells were cotransfected with an ORF8-encoding plasmid together with a plasmid encoding spike protein with the indicated mutations. The cells were collected at 3 days post-transfection and subjected to immunoprecipitation assays. Immunoblotting analysis of the total cell lysates and the immunoprecipitates were performed using antibodies against the spike protein, FLAG tag, and actin. Abbreviations of Spike mutants: A: d69-70; D: T19R; G: L18F/T20N/P26S. **(D)** Spike pseudovirions were produced using a plasmid encoding different spike envelopes together with the lentiviral vector pLAS-IRES-GFP (EV) or pLAS-ORF8-IRES-GFP (ORF8). After purification of the pseudovirions, their transduction efficiency was evaluated by exposing HEK293T/hACE2 cells to the same amount of viral supernatant. The percentages of GFP-positive cells the transduced cells were evaluated by flow cytometry and normalized with EV d69-70 sample in each experiment. **(E)** The relative quantity of Spike pseudoviruses in the same samples shown in **(D)** was evaluated by a binding assay of HEK293T/hACE2 cells exposed to the same volume of different viral samples, and samples were then visualized with a polyclonal antibody recognizing the spike protein and a Cy3-conjugated secondary antibody. DAPI was used for staining nuclei. The scatter plot figures in **(A,B,D)** show the mean ± SD (error bars). Unpaired Student’s *t*-test was used in **(A,B)** and paired Student’s *t*-test was used in **(D)**. *p* < 0.05 indicates a statistically significant difference; *p < 0.05, ***p < 0.001. The scale bar represents 100 μm.

We showed that ORF8 had a decreased binding ability to the mutant spike proteins with the deletion at the N-terminus from amino acids 35 to 102 or 528 to 685 (Figure 3C). Indeed, these two regions are also reportedly enriched with mutations identified in SARS-CoV-2 variants with elevated transmission characteristics (Harvey et al., 2021; Zhou and Wang, 2021). We are particularly interested in the mutations at the N-terminus because their functions have been less reported than those of mutations in the RBD and S1/S2 cleavage domains. We constructed spike mutations based on N501Y/D614G together with the 69–70 deletion (observed in B1.1.7; SARS-CoV-2 Alpha), T19R (observed in B.1.617.2; SARS-CoV-2 Delta), or L18F/T20N/P26S (observed in P.1; SARS-CoV-2 Gamma; Harvey et al., 2021) and examined the impact of ORF8 on these spike variants in the transfected cells. The flow cytometry analyses of surface spike protein showed that some of these spike variants gained higher surface spike protein expression than control spike under the expression of ORF8 while this phenomenon might mostly be due to the higher expression of spike protein in these variants but not gained the resistance to ORF8-mediated spike protein downregulation (Figures 6A,B). Indeed, the immunoprecipitation assay revealed that these spike variants shared the similar ability to bind ORF8 (Figure 6C). Ultimately, we observed that Spike pseudoviruses baring spike variant with 69–70 deletion had a significantly higher transduction rate accompanying by more viral particles, but we did not observe the rescue of transduction rate by any of the spike variant after the expression of ORF8 during Spike pseudoviral production (Figures 6D,E; Supplementary Figure 6).

## Discussion

RNA-dependent RNA polymerase found in RNA viruses commonly causes error-prone replication, which contributes to the adaption or evolution of RNA viruses to their hosts (Duffy et al., 2008; Simmonds et al., 2019; Grubaugh et al., 2020). Ultimately, the RNA viruses that acquire mutations that benefit viral spreading could rapidly become the dominant during viral evolution (Domingo and Holland, 1997; Duffy et al., 2008; Simmonds et al., 2019). One very good example is the D614G mutation in the spike protein of SARS-CoV-2, and the variant with this mutation became dominant within just a few months after the COVID-19 outbreak because this point mutation moderately increases viral titer and fitness advantage that benefits the transmission (Hou et al., 2020; Korber et al., 2020; Plante et al., 2021). However, many other factors might also contribute to viral evolution during human-to-human transmission, and these factors include the requirement for immune evasion from innate and adaptive immunity, increased efficiency of viral replication, elevated efficiency of viral packaging, and immune escape and protection from currently available vaccines (Duffy et al., 2008; Simmonds et al., 2019). Nevertheless, many of these hypotheses, particularly the precise functions of each viral protein during the lifecycle, require fundamental virology studies based on classical viral mutations or deletions at specific genes, whereas many factors, such as the requirement of biosafety level 3 laboratories, high viral mutation rates, the difficulty and risks associated with obtaining recombinant virus and generating mutations, and the difficulty in studying the functions of each viral protein using the siRNA knockdown system for RNA viruses, restrain the progress of these studies on SARS-CoV-2. Mutations in the spike protein have been carefully checked because this protein forms the outermost layer of SARS-CoV-2, determines the receptor binding and fusion of the virus, and thus directly contributes to transmission (Harvey et al., 2021; Zhou and Wang, 2021). Many spike-pseudovirus may be applied without the usage of real viruses, which further facilitates this study. Nevertheless, many of the mutation sites in spike proteins acquired from highly transmitted variants remain unknown (Harvey et al., 2021).

Here, we investigated the roles of the ORF8 protein, which is encoded by the most hypervariable gene among betacoronaviruses and many clinical variants of SARS-CoV-2 (Ceraolo and Giorgi, 2020; Lu et al., 2020; Tan et al., 2020; Alkhansa et al., 2021), in modulating viral transmission and focused on its roles in regulating the spike protein. We found that the ORF8 protein of SARS-CoV-2 could modulate the protein level and processing of the spike protein in a dose-dependent manner, which might lead to a decrease in viral spreading observed with the spike-pseudovirus. The interaction of ORF8 with the spike protein could strongly lead to modulation of the expression and processing of the spike protein. Some ORF8 variants showed a reduced ability to modulate the spike protein by downregulating their stability, which may contribute to an increase in spike protein expression without compromising the roles of ORF8 in downregulating the expression of HLA-A2 (Supplementary Figures 5B,C). We further found that certain spike mutants with deletions at specific regions were resistant to the inhibitory effects of ORF8, and interestingly, these regions were reported as hot spot regions during spike evolution. Ultimately, we found that some spike variants with high transmission ability showed higher resistance to ORF8-induced inhibitory effects. Altogether, our data suggest that ORF8 is involved in viral transmission by exerting negative effects on the level and cleavage of spike proteins. Meanwhile, our studies on the clinical variants of ORF8 and spike proteins further showed the possibility that these variants might take advantage of viral transmission by reducing the inhibitory effects of ORF8 on the spike protein.

In addition to the ORF8 variants identified in clinical isolates, the deletion of ORF8 variants of SARS-CoV-2 was reported at the early stage of the COVID-19 outbreak in Singapore, where 23.6% of the screened samples showed the deletion of ORF8 (Su et al., 2020). Similar ORF8-deletion variants of SARS-CoV-2 have also been reported in Taiwan, Australia, Bangladesh, Spain, and other geographic areas (Gong et al., 2020; Young et al., 2020; Zinzula, 2021). Interestingly, individuals carrying ORF8-deletion SARS-CoV-2 variants exhibit milder clinical symptoms and better outcomes but similar viral loads as individuals infected with the wild-type virus (Gong et al., 2020; Su et al., 2020; Young et al., 2020; Zhou and Wang, 2021). This phenomenon could be explained by the later study validating that overexpression of ORF8 in the lung leaded to cytokine storm through IL-17 pathway in animal model (Lin et al., 2021). In contrast, the SARS-CoV-2 ORF8 of the 382-nt deletion variant shows significantly higher replicative fitness *in vitro* than that of the wild-type virus, but no significant changes in viral replication ability were found between these two viruses (Gong et al., 2020; Su et al., 2020); these *in vitro* data indicate that the deletion of ORF8 in SARS-CoV-2 might contribute to an increase in the viral titer *in vitro* by increasing viral packaging. Although ORF8 deletion SARS-CoV-2 variants have been reported in many cases, these ultimately did not become dominant variants during the pandemic spreading of SARS-CoV-2, and this finding may indicate the importance of ORF8 in human-to-human viral transmission. For example, ORF8 could contribute to immune evasion by downregulating MHC-I and lead to reduced T cell cytotoxicity against virus-infected cells (Zhang et al., 2021). Moreover, the intracellular aggregates of ORF8 in human cells may lead to the inhibition of IFNβ- or IFNγ-induced antiviral gene expression (Li et al., 2020; Geng et al., 2021; Rashid et al., 2021). Nevertheless, most of the infected individuals during the COVID-19 pandemic are healthy individuals with competent immune responses. These immunosurveillance functions of ORF8 might explain why ORF8 deletion variants could not become the dominant variants during the COVID-19 pandemic even though they could achieve increased viral spreading by up to 100-fold, as shown *in vitro* in Vero-E6 cells previously (Su et al., 2020).

Because ORF8 exerts double-edged effects on viral transmission and immune evasion, one possible consequence of the evolution of SARS-CoV-2 is that the mutations acquired by ORF8 reduce its inhibitory effects on the spike protein but maintain the immune escape ability of the virus. Alternatively, the spike protein has gained mutations that contribute to reductions in the inhibitory effects of ORF8. Since the spike protein and MHC-I protein are critical for viral transmission and the immunity of the host cells and both of them can be modulated by ORF8, we hereby wish to investigate the downregulating effects of different ORF8 mutations on these two proteins in parallel. Our findings showed that the ORF8 L84S mutation exerted weaker inhibitory effects on the spike protein through a reduction in ORF8 stability but did not significantly affect the inhibition of MHC-I expression. We also wish to investigate whether the dimerization or polymerization of ORF8 is essential for its ability to downregulate the spike protein or even MHC-I, but we failed to obtain an ORF8 mutant that was unable to undergo dimerization with ORF8 mutants carrying C20A or 73YIDI76 deletion. The reported regions might play important roles in ORF8 dimerization (Flower et al., 2021).

Moreover, we found that some mutant spike proteins with specific deleted regions were resistant to ORF8-induced downregulation. We observed that spike proteins with deletions in the N-terminal domain (amino acids 35–132) or S1/S2 protein cleavage region (amino acids 528–685) were less physically interacted with ORF8. Interestingly, mutations in both regions have been observed in the clinical SARS-CoV-2 variants that prevail in the current COVID-19 pandemic. Importantly, the functions of spike variants carrying mutations at the N-terminal region of the NTD, such as resulting in alterations in conformation, decreasing the neutralization of NTD-specific neutralizing antibodies, or exerted a lower effect on glycosylation, remain controversial (Harvey et al., 2021). Nevertheless, RBD-binding antibodies still show dominant roles in neutralizing SARS-CoV-2 infection, and the impact of the mutations within this region should be investigated (Piccoli et al., 2020; Harvey et al., 2021). Interestingly, our data showed that spike proteins with mutations at N-terminal regions reported by the highly transmissible SARS-CoV-2 variants were indeed more resistant to ORF8-mediated downregulation. Our data might explain why SARS-CoV-2 acquires these mutations during its viral evolution.

The mechanisms through which ORF8 downregulates the spike protein were also investigated at the protein level in our study. We excluded the possibility that ORF8 regulates the spike protein at the transcriptional level because both ORF8- and spike-encoding plasmids were driven by the CMV promoter. Moreover, the inhibitory effects of ORF8 can also be observed in the expression plasmid encoding the codon-optimized spike gene (data not shown). By testing many proteinase inhibitors, we observed that the inhibitory effects of ORF8 were hardly recovered in the samples transfected with ORF8-encoding plasmids at high amounts (spike to ORF8 ratio of 1:9). However, we did observe the recovery of the spike protein on the surface of the cells transfected with a lower amount (spike to ORF8 ratio of 1:3) of ORF8-encoding plasmid using different proteinase inhibitors targeting autophagy, proteasome, ER-associated protein degradation, and lysosomal degradation. Our results also showed that DBeQ and NH_4_Cl exerted strong effect on elevating surface spike protein expression in cells overexpressing ORF8 but did not have significant influence on the expression of MHC-I. Importantly, the recently published interactome data of SARS-CoV-2 viral proteins suggests that ORF8 is highly associated with many ER-related proteins, which may explain the effects of DBeQ observed in our study (Gordon et al., 2020). These results suggest that ORF8 might contribute to reductions in the spike protein through multiple mechanisms, which might explain the difficulty in recovering the spike protein expression level to a similar level where ORF8 protein is presented in the cells. A detailed investigation of ORF8 and thus a full understanding of this protein might contribute to the treatment of SARS-CoV-2.

Studying clinically isolated SARS-CoV-2 variants that clearly show advantages in transmission and clinical symptoms is crucial for understanding the roles of viral proteins in the viral lifecycle, immune evasion, and transmission. Nevertheless, SARS-CoV-2 is an RNA virus with a giant genome, and many mutations at different genes co-exist in the same variant, which makes it extremely difficult to unveil the functions of these variants at each gene level. Mutations in the spike protein can be examined using a pseudovirus platform, whereas elucidating the molecular mechanisms of each viral protein and its variations at each step of viral life cycle seems extremely difficult from clinical isolated viruses. The recombinant SARS-CoV-2 BACmid system, infectious cDNA clone, or replicon may help researchers elucidate the functions of each gene and even study the functions of each point mutation at each specific protein in an clean genetic background (Xie et al., 2020, 2021; Ye et al., 2020; He et al., 2021; Kotaki et al., 2021; Rihn et al., 2021) without influences contributed by the other mutations co-exist at the other viral proteins.

In summary, here we report the strong potency of ORF8 in suppressing the expression and the cleavage of the spike protein that may consequently influence the amount of the processed spike protein used for viral package. We also observed that ORF8 interacted with S1 domain of the spike protein and downregulated the spike protein through multiple proteolytic pathways. Several ORF8 and spike protein mutations identified from clinical samples were also examined. We observed that ORF8 with the mutations at L84S or C20A were less potent in modulating the spike protein and with higher protein turnover rate. In parallel, the inhibition effects of ORF8 on the spike protein with mutations observed in highly transmissible SARS-CoV-2 variants can be also validated. This work provides a basis for further studies to determine the crosstalk between different viral proteins in SARS-CoV-2 virology and the possible mechanisms in its transmission.

## Data Availability Statement

The original contributions presented in the study are included in the article/Supplementary Material, and further inquiries can be directed to the corresponding author.

## Author Contributions

M-HT designed the study and wrote the manuscript. M-HT, J-MC, J-LT, J-NH, and I-HC performed the experiments. M-HT, J-MC, J-LT, and S-TC analyzed the data. All authors contributed to the article and approved the submitted version.

## Funding

This work was supported by the Ministry of Science and Technology (MOST) of Taiwan (grant numbers MOST109-2327-B-010-005 and MOST111-2321-B-A49-007) and by the Excellent Youth Scholar Grant and Young Scholar Fellowship (Columbus) Program (grant numbers MOST109-2636-B-010-007 and MOST110-2636-B-A49-002).

## Conflict of Interest

The authors declare that the research was conducted in the absence of any commercial or financial relationships that could be construed as a potential conflict of interest.

## Publisher’s Note

All claims expressed in this article are solely those of the authors and do not necessarily represent those of their affiliated organizations, or those of the publisher, the editors and the reviewers. Any product that may be evaluated in this article, or claim that may be made by its manufacturer, is not guaranteed or endorsed by the publisher.
