# Supplementary material for: The ORF8 Protein of SARS-CoV-2 Modulates the Spike Protein and Its Implications in Viral Transmission
Source: Front Microbiol. 2022 May 19;13:883597. doi: 10.3389/fmicb.2022.883597 (PMC9161165; doi:10.3389/fmicb.2022.883597)
Supplement: Supplementary file 1 [file Data_Sheet_1.docx]

The ORF8 protein of SARS-CoV-2 modulates the spike protein and its implications in viral transmission

Jen-Mei Chou^1, 2*^, Jo-Ling Tsai^1,2*^, Jo-Ning Hung^1, 2^, I-Hua Chen^1, 2, 3^,

Szu-Ting Chen^4, 5^, & Ming-Han Tsai^1, 2, 5#^

**Supplemental Material**

**Supplementary Figures 1-6**

**Supplementary Table 1**

**Supplementary Figures**


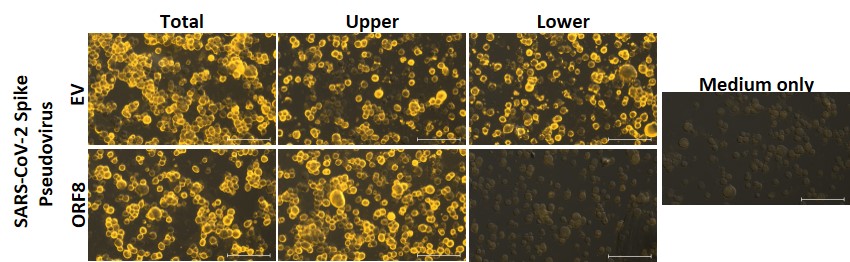


**Figure S1: Evaluation of viral-free spike protein and Spike pseudoviruses in the supernatants produced from HEK293T cells.** The cell culture supernatants collected for the purification of Spike pseudoviruses as described in Fig. 1 were subjected to centrifugation to concentrate the virus. The total supernatants before centrifugation (total), the upper layer of the supernatants after centrifugation (waste), and the purified pseudovirions (lower) were used for a binding assay by exposure to HEK293T/hACE2 cells as described in Fig. 1. The same amount of supernatant was used in the binding assay. The concentrated Spike pseudoviruses were diluted in medium to the original volume before use in the binding assay. The spike protein or Spike-bearing pseudovirions bound to the cells were further visualized through immunofluorescence staining by using an antibody recognizing the spike protein. The scale bar represents 100 μm.


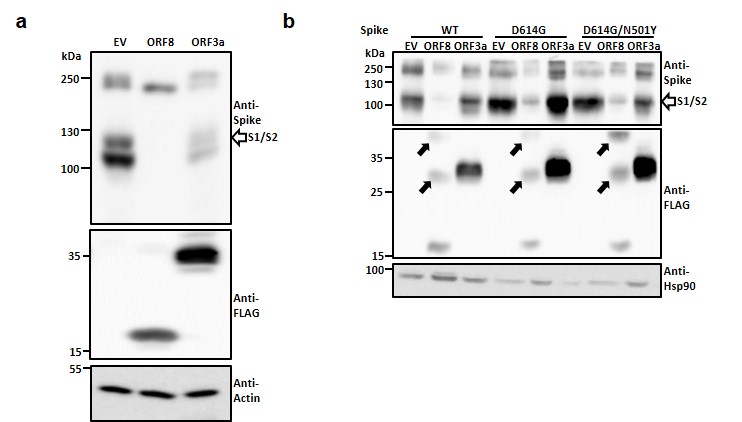


**Figure S2: Evaluation the effects of C9-tag fused with the spike protein and the dimerization of ORF8.** (a) A plasmid encoding the spike protein without C9 fusion tag at its C-terminal was applied for the same experiment shown in Fig. 2b. Here an antibody against actin serves as control. (b) The samples shown in Fig. 2c were prepared under nondenaturing conditions and subjected to SDS-PAGE for immunoblotting. The arrows indicate dimerization or polymerization of the ORF8 protein.


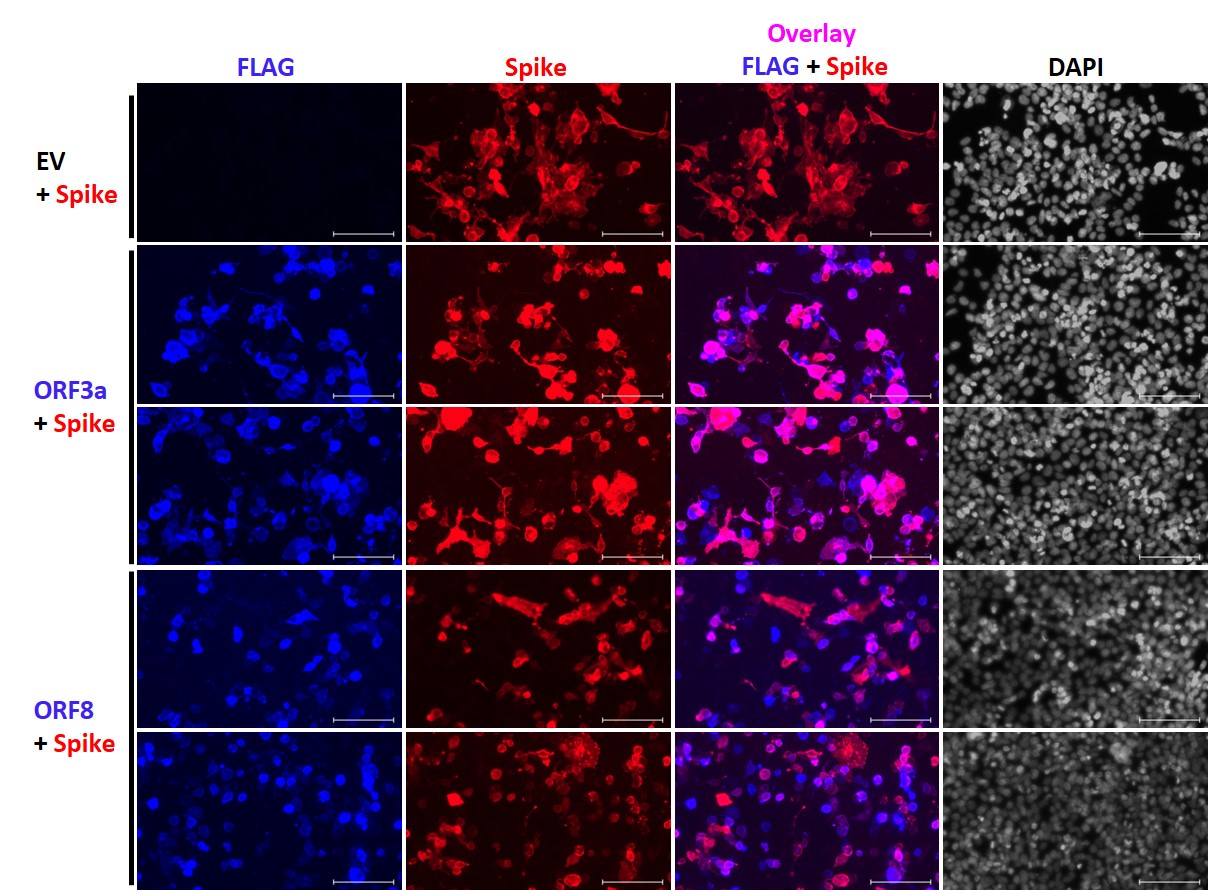


**Figure S3: Impact of ORF8 on the downregulation of the spike protein.**

Immunofluorescence staining of HEK293T cells cotransfected with a plasmid encoding spike protein together with a plasmid encoding either ORF8-FLAG or ORF3a-FLAG protein at 4 days post transfection and visualized by antibodies against spike and FLAG. DAPI was used as counterstain. The scale bar represents 100 μm.

**
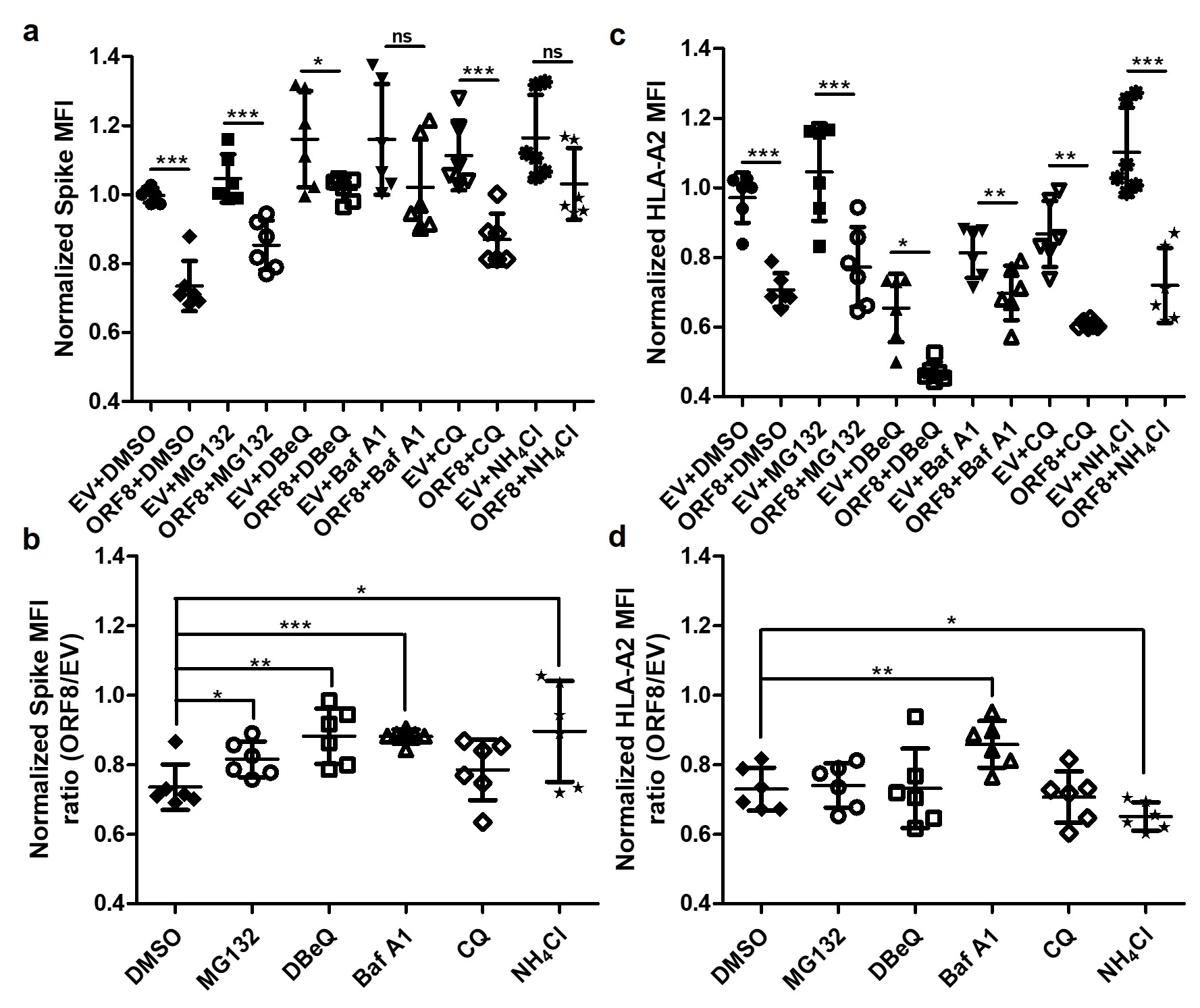
Figure S4: Evaluation of the effects of different inhibitors on ORF8-mediated downregulation of the spike protein and HLA-A2.** (a and b) Same experiments shown in Fig. 4b but here the expression levels of the surface (a) spike protein and (b) HLA-A2 were shown; including the EV-transfected cells after the treatment of different inhibitors in the same experiments. (c and d) The expression level of the surface (c) spike protein and (d) HLA-A2 in between EV- or ORF8-transfected cells were further calculated as ratio format. All figures show the means ± SDs (error bars) and unpaired Student’s *t* test was used, and *P* < 0.05 indicates a statistically significant difference; * *P* < 0.05, ** *P* < 0.01, *** *P* < 0.001.


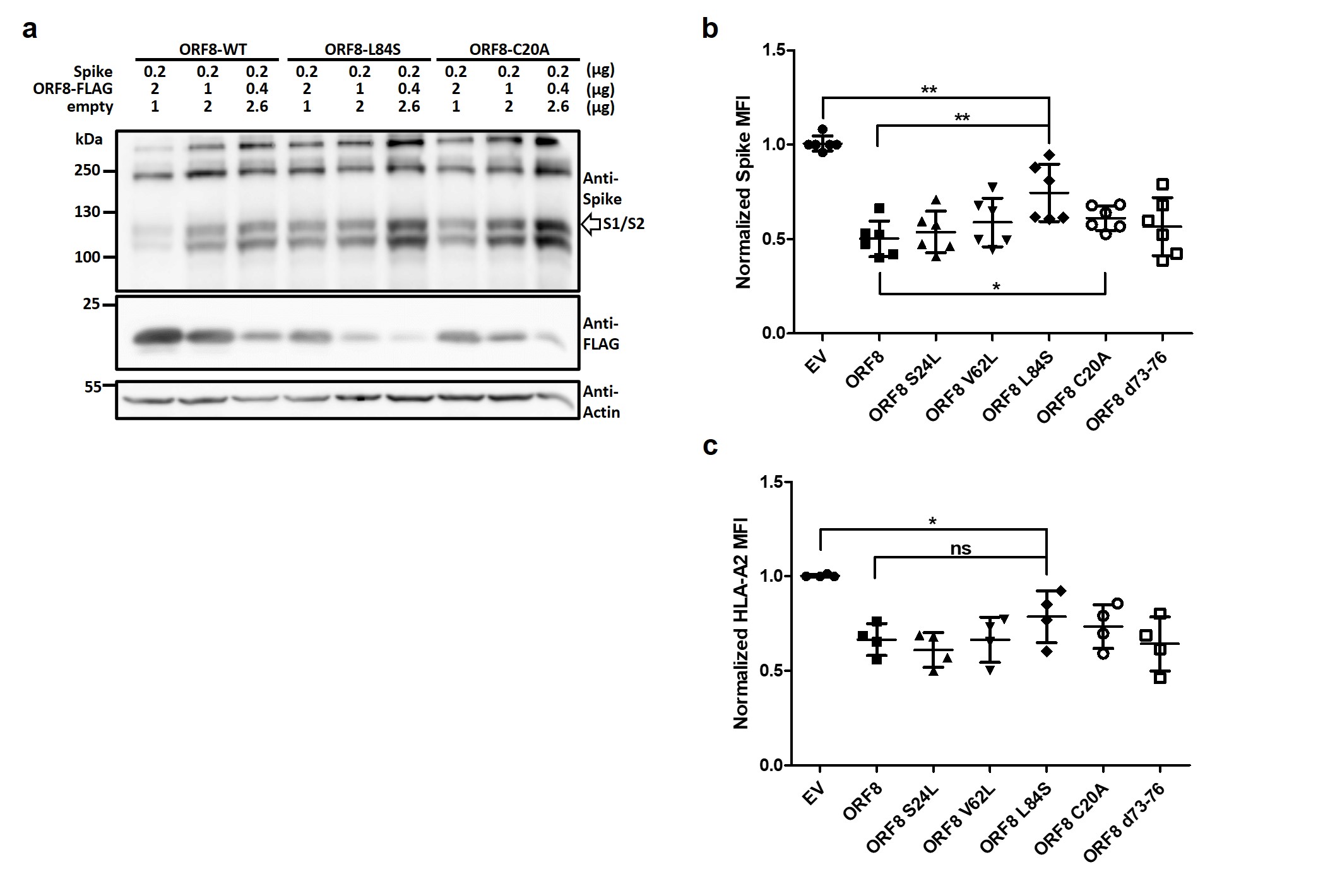


**Figure S5: Impact of ORF8 mutations on regulating the spike protein, their dimerization, and the MHC-I levels.**

(a) HEK293T cells were transfected with a plasmid encoding the spike protein together with a plasmid encoding ORF8 or its mutants at different amounts. Three days post transfection, we performed immunoblotting analyses using antibodies against FLAG, the spike protein and actin. (b and c) HEK293T cells transfected with a plasmid encoding the spike protein and a plasmid encoding the indicated ORF8 variants at 3 days post transfection were analysed by flow cytometry after staining with antibodies against (b) the spike protein and (c) HLA-A2. All figures show the means ± SDs (error bars) and unpaired Student’s *t* test was used, and *P* < 0.05 indicates a statistically significant difference; * *P* < 0.05, ** *P* < 0.01, ns: no significance.


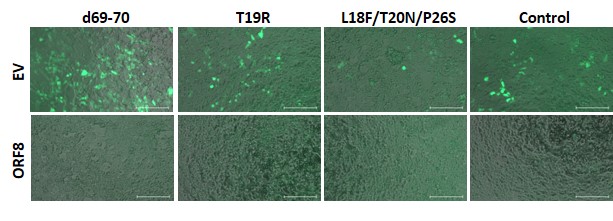


**Figure S6: Influence of spike mutations on ORF8-mediated downregulation of the spike protein.**

Spike pseudoviruses were produced using the pLAS2-IRES-GFP (EV) or pLAS2-ORF8-IRES-GFP (ORF8) lentiviral vector incorporated with the indicated spike mutants. After the purification of these pseudoviruses, an equal amount of viral supernatant was used to test their transduction efficiencies in HEK293T/hACE2 cells, which were observed as GFP-positive cells 4 days post transduction and visualized by fluorescence microscopy. We showed the cells transduced with less amount of viruses to emphasize the different transduction rate in between each group. The scale bar represents 200 μm.

**Supplementary Table 1. Oligonucleotides used in the study.**

| **Name** | **Sequence 5'-3'** | **Aim** |
| --- | --- | --- |
| BGH rev | tagaaggcacagtcgagg | Overlapping PCR in pcDNA 3.1 |
| PEGFP-N5 fwd | taggcgtgtacggtgggagg |  |
| pLAS2 fwd | tttgctagcccgggatccacagggggatcgtagtcgac | PCR cloning of ORF8 and ORF3a into pLAS2 plasmid |
| pLAS2 rev | tttacgcgtgatatcgaattcttacttgtacagctcgtccatg |  |
| D614G fwd | ggccgtgctgtaccagggtgtgaattgcaccgagg | spike mutation in D614G |
| D614G rev | cctcggtgcaattcacaccctggtacagcacggcc |  |
| N501Y fwd | tacggcttccagccaacctacggagtgggataccagc | spike mutation in N501Y |
| N501Y rev | gctggtatcccactccgtaggttggctggaagccgta |  |
| Spike C-del+C9 fwd | ﻿gacctgaccgagacatcccaggtcgctcccgcttagc | spike mutation in del 820-1271 a.a. |
| Spike C-del+C9 rev | tcgagctaagcgggagcgacctgggatgtctcggtcag |  |
| RBD fwd | ﻿aagtgcaccctgaagagcaagaagagcaccaatctg | spike mutation in del 306-527 a.a. |
| RBD rev | ﻿cagattggtgctcttcttgctcttcagggtgcactt |  |
| S1/S2 fwd | ﻿gctaccgtgtgcggaccaagcgtggccagccagtcc | spike mutation in del 528-685 a.a. |
| S1/S2 rev | ﻿ggactggctggccacgcttggtccgcacacggtagc |  |
| HV69-70del fwd | gacctggttccacgccatcagcggcaccaatggcacca | spike mutation in del 69-70 a.a. |
| HV69-70del rev | tggtgccattggtgccgctgatggcgtggaaccaggtc |  |
| GSAS fwd | gacccagaccaactccccagggtctgcttccagcgtggccagccagtcca | spike mutation in GSAS |
| GSAS rev | tggactggctggccacgctggaagcagaccctggggagttggtctgggtc |  |
| T19R fwd | ctcagtgcgtgaacctgagaactagaacccagctgcctcct | spike mutation in T19R |
| T19R rev | aggaggcagctgggttctagttctcaggttcacgcactgag |  |
| LTP fwd | ttcactaatagaacccagctgccttctgcctatactaactccttc | spike mutation in L18F/T20N/P26S |
| LTP rev | aaggcagctgggttctattagtgaagttcacgcactgagacgag |  |
| S1 del fwd | gctgcctctggtctcgtctagcgtggccagccagtccatc | spike mutation in del 14-685 a.a. |
| S1 del rev | gatggactggctggccacgctagacgagaccagaggcagc |  |
| D614G fwd | ggccgtgctgtaccagggtgtgaattgcaccgagg | spike without C9 tag |
| Spike rev | tagactcgagctatgtatagtgcagtttgac |  |
| ORF8 S24L fwd | caggaatgcagcctacaactgtgtacccagcaccagccgt | ORF8 mutation in S24L |
| ORF8 S24L rev | acggctggtgctgggtacacagttgtaggctgcattcctg |  |
| ORF8 V62L fwd | cactcattgagttgtgtctggacgaggccggttca | ORF8 mutation in V62L |
| ORF8 V62L rev | tgaaccggcctcgtccagacacaactcaatgagtg |  |
| ORF8 L84S fwd | gaaattacactgtctcctgtagccccttcaccatcaactg | ORF8 mutation in L84S |
| ORF8 L84S rev | cagttgatggtgaaggggctacaggagacagtgtaatttc |  |
| ORF8C20A-fwd | gcagcttttcatcaggaagccagcctacaatcgtgtac | ORF8 mutation in C20A |
| ORF8C20A-rev | gtacacgattgtaggctggcttcctgatgaaaagctgc |  |
| ORF8 73-76del fwd | ﻿caaaatccccaatccagggaaattacactgtctc | ORF8 mutation in del 73-76 a.a. |
| ORF8 73-76del rev | ﻿gagacagtgtaatttccctggattggggattttg |  |
